# Olfactory subsystems in the honeybee: sensory supply and sex specificity

**DOI:** 10.1007/s00441-014-1892-y

**Published:** 2014-05-13

**Authors:** Jan Kropf, Christina Kelber, Kathrin Bieringer, Wolfgang Rössler

**Affiliations:** 1 Department of Behavioral Physiology and Sociobiology, Biozentrum, University of Würzburg, Am Hubland, 97074 Würzburg, Germany; 2 Ecological Networks, Technical University of Darmstadt, Schnittspahnstrasse 3, 64287 Darmstadt, Germany

**Keywords:** *Sensilla basiconica*, Antennal lobe, Glomeruli, Projection neuron, Honeybee drone

## Abstract

The antennae of honeybee (*Apis mellifera*) workers and drones differ in various aspects. One striking difference is the presence of *Sensilla basiconica* in (female) workers and their absence in (male) drones. We investigate the axonal projection patterns of olfactory receptor neurons (ORNs) housed in *S. basiconica* in honeybee workers by using selective anterograde labeling with fluorescent tracers and confocal-microscopy analysis of axonal projections in antennal lobe glomeruli. Axons of *S. basiconica*-associated ORNs preferentially projected into a specific glomerular cluster in the antennal lobe, namely the sensory input-tract three (T3) cluster. T3-associated glomeruli had previously been shown to be innervated by uniglomerular projection (output) neurons of the medial antennal lobe tract (mALT). As the number of T3 glomeruli is reduced in drones, we wished to determine whether this was associated with the reduction of glomeruli innervated by medial-tract projection neurons. We retrogradely traced mALT projection neurons in drones and counted the innervated glomeruli. The number of mALT-associated glomeruli was strongly reduced in drones compared with workers. The preferential projections of *S. basiconica*-associated ORNs in T3 glomeruli together with the reduction of mALT-associated glomeruli support the presence of a female (worker)-specific olfactory subsystem that is partly innervated by ORNs from *S. basiconica* and is associated with the T3 cluster of glomeruli and mALT projection neurons. We propose that this olfactory subsystem supports parallel olfactory processing related to worker-specific olfactory tasks such as the coding of colony odors, colony pheromones and/or odorants associated with foraging on floral resources.

**Electronic supplementary material:**

The online version of this article (doi:10.1007/s00441-014-1892-y) contains supplementary material, which is available to authorized users.

## Introduction

Olfaction is an ancient sensory modality and plays a crucial role in most animals for approaching or avoiding various odor sources and for judging their quality in a variety of behavioral contexts. Whereas odorant reception at the molecular level exhibits distinct differences between vertebrates and insects, the basic wiring pattern of receptor neurons with second-order neurons within the primary olfactory centers, the vertebrate olfactory bulb and the insect antennal lobe (AL) shows several striking similarities. These have been the subject of intense research over recent years (for reviews, see Hildebrand and Shepherd 1997; Ache and Young 2005; Touhara and Vosshall 2009; Martin et al. 2011).

Insect antennae are covered with various types of sensory sensilla; most of them being specialized for chemoreception but also for hygro-, mechano- and thermoreception (Esslen and Kaissling 1976; Maronde 1991; Ai et al. 2007). Olfactory sensilla house the olfactory receptor neurons (ORNs) that extend axons into spheroidal structures termed glomeruli to form synaptic connections with local interneurons and projection neurons (PNs). Glomeruli represent the functional units of the AL (e.g., Anton and Homberg 1999). In the honeybee, *Sensilla placodea*, *Sensilla trichoidea* and *Sensilla basiconica* have been classified as olfactory sensilla, either according to their odor-response profiles in single-sensillum recordings (*S. placodea*) or based on specific anatomical features (*S. trichoidea*, *S. basiconica*; Lacher 1964; Esslen and Kaissling 1976; Akers and Getz 1993; Getz and Akers 1993). ORN axons from olfactory sensilla project via four distinct AL sensory-input tracts to four clusters of glomeruli in the AL termed the T1-T4 cluster (Mobbs 1982; Galizia et al. 1999; Abel et al. 2001; Kirschner et al. 2006). Axons from ORNs in *S. placodea* project to all four clusters of glomeruli T1-T4 (Brockmann and Brückner 1995; Kelber et al. 2006) and single-sensillum recordings from *S. placodea* have revealed responses to a broad range of odorants (Getz and Akers 1993). This might be either caused by the broad tuning of ORNs or attributable to the finding that *S. placodea* house many individual ORNs (between 7 and 23; Kelber et al. 2006), each covering a certain spectrum of molecular receptive ranges.

In insects, PNs convey the olfactory information to the mushroom bodies (MBs), higher sensory association centers and sites associated with learning and memory (Gerber et al. 2004; Davis 2005; Giurfa 2007; Hourcade et al. 2010; Cervantes-Sandoval et al. 2013). Uniglomerular PNs in the honeybee and other Hymenoptera have been shown to project to the MBs and lateral horn (LH) via two parallel tracts: the medial and the lateral AL tracts (mALT and lALT) forming a dual olfactory pathway (Abel et al. 2001; Kirschner et al. 2006; Galizia and Rössler 2010; Rössler and Brill 2013; tract terminology according to Ito et al. 2014). Comparative anatomical studies indicate that a dual olfactory pathway probably emerged in the basal Hymenoptera (Dacks and Nighorn 2011; Rössler and Zube 2011; Rössler and Brill 2013). However, the selective pressure that promoted the evolution of a dual olfactory pathway within this group of insects remains to be further investigated (Rössler and Brill 2013).

Female honeybee workers show complex social behavior that is largely influenced by pheromonal communication (Slessor et al. 2005; Le Conte and Hefetz 2008). This is different in male drones, which mainly perform reproductive tasks, do not forage actively for food and might not need to distinguish minor changes in colony pheromone concentrations. On the other hand, drones are highly sensitive to the queen sex-pheromone (Gary 1962). Therefore, differences in the olfactory system reflecting these behavioral specializations are likely to exist between honeybee workers and drones. One striking sex-specific difference is the absence of *S. basiconica* on drone antennae (Esslen and Kaissling 1976; Nishino et al. 2009). Furthermore, drone ALs contain a smaller number of glomeruli compared with both female castes (workers and queens) but comprise several enlarged macroglomeruli (Arnold et al. 1985; Sandoz 2006; Groh and Rössler 2008; Nishino et al. 2009). The largest macroglomerulus has been shown to respond to the major component of the queen mandibular pheromone (Sandoz 2006; Wanner et al. 2007). The reduction of AL glomeruli is mostly associated with the T3 cluster (Nishino et al. 2009), which has been demonstrated to be mainly innervated by medial tract PNs in honeybee workers (Kirschner et al. 2006). Comparative studies in other Hymenoptera indicate that the lack of *S. basiconica* in males is a characteristic trait across both social and solitary Hymenoptera (Ågren 1977, 1978; Wcislo 1995; Ågren and Hallberg 1996; Nakanishi et al. 2009, 2010; Mysore et al. 2010; Nishikawa et al. 2012; Streinzer et al. 2013). In the leaf-cutting ant *Atta vollenweideri*, *S. basiconica* have been found exclusively to innervate a specific (T6) cluster of AL glomeruli (Kelber et al. 2010).

The absence of *S. basiconica*, together with the reduction of glomeruli in the T3 cluster, in honeybee drones suggests that ORNs from *S. basiconica* preferentially innervate glomeruli in the T3 cluster and are associated with medial-tract PNs. To test this hypothesis, we investigated the axonal projections of ORNs in the hair-like olfactory antennal sensilla of female worker bees, with a special focus on *S. basiconica* and, in particular, their glomerular innervation patterns and their association with PN output tracts. Furthermore, we retrogradely labeled the mALT in drones to analyze whether glomeruli associated with this tract are reduced.

## Materials and methods

### Animals

Honeybee workers and drones (*Apis mellifera carnica*) were collected from bee hives of the institutional bee station at the University of Würzburg. Workers were taken from the entrance of the hive and drones were caught directly in the hive. In both cases we did not control for age. The animals were cooled in a refrigerator (4 °C), fixed in custom made plastic holders and provided with sugar solution (40 %) ad libitum.

### Staining of axonal projections

**Fig. 1:**
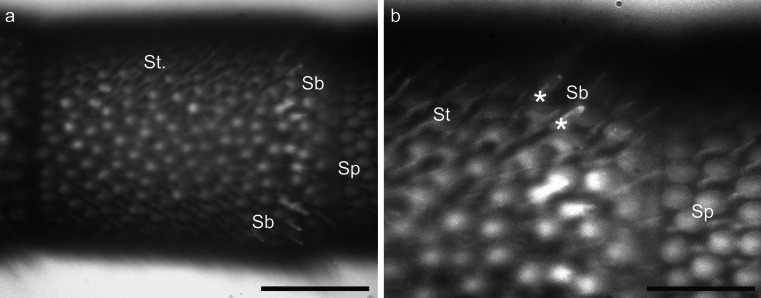
Light microscopic images of *Sensilla basiconica* (*Sb*), *Sensilla placodea* (*Sp*) and *Sensilla trichoidea* (*St.*, *St*) on a honeybee worker antenna. **a** Overview of the ninth segment. *Bar* 100 μm. **b** Detailed view of the *S. basiconica*-rich region on the ninth segment (*white asterisks* bases of two identifiable *S. basiconica*). *Bar* 25 μm

ORNs from hair-like sensilla on defined antennal segments were labeled by using a method previously described by Kelber et al. (2010). Cut glass microelectrodes were placed close to a defined segment of the antenna under visual control (280×) by using a photo-microscope (M 400, Wild, Heerbrugg, Germany) or an Olympus imaging system (200×-400×, upright microscope: BX51WI, filter set: U-MF2 excitation 395/440 FT 460 emission 540/50, objective: XLUMP, NA 0.95, light source: MT20, software: Cell R v2.5 [all Olympus Imaging Europa], camera: model 8484-03G [Hamamatsu Photonics]) and a micromanipulator (Junior Unit, Luigs & Neumann, Ratingen, Germany). This setup allowed the identification of individual sensilla (Fig. 1a, b). The electrode was mounted on a piezo element (Element, EPZ-Serie, Conrad Electronic, Hirschau, Germany) connected to a function generator (PM 5133, Fluke/Philips, Kassel, Germany). Vibration (2-13 kHz) of the piezo element and therefore also of the pipette was used to cut the hair-like sensilla and to expose the dendrites of the ORNs within sensilla of a defined antennal segment. Subsequently, a droplet of Biotin Dextran (Molecular Probes, D-7135, Leiden, Netherland) or Microruby (tetramethylrhodamine dextran with biotin, 3,000 MW, lysine-fixable, D-7162; Molecular Probes, Eugene, Ore., USA), 3-5 % in distilled water, was applied onto the remaining sensilla stumps to enable the dye to enter the ORN dendrites. This method allowed selective staining of hair-like sensilla (*S. trichoidea*, *S. basiconica*) and excluded the staining of *S. placodea*, as these sensilla are located beneath a plate-like structure of the antennal cuticle (Esslen and Kaissling 1976; Kelber et al. 2006). Animals were then kept in the dark for 24 h before the brains were dissected. For the staining of single sensilla, a microelectrode was placed close to one sensillum to cut only a single sensillum hair. Because of their small size, the cutting of individual basiconic sensilla was extremely difficult and the success rate for staining was extremely low (only one staining after the approved cut of only one *S. basiconicum*). Therefore, in most cases, we moved the electrode, in particular in *S. basiconica*-rich regions close to the borders of antennal segments, to cut small groups of hair-like sensilla preferentially including *S. basiconica* (Fig. 1a, b). Larger numbers of *S. basiconica* could be found in ring-like arrangements in the distal region of the antennal segments 3-10, close to the transition to the next segment (Fig. 1; Esslen and Kaissling 1976; Nishino et al. 2009). These axonal projections in this staining technique were compared with the staining of ORN axons from *S. trichoidea* only. Larger groups of *S. trichoidea* in the middle of antennal segments that lacked any *S. basiconica* (Fig. 1) were cut with a sharp tungsten wire (diameter: 200 μm) attached to the piezo element. Microelectrodes used in all experiments (including mALT staining; see below) were pulled from thin-walled glass pipettes (1B100F-3, WPI, Sarasota, USA) with a DMZ-Universal Puller (Zeitz-Instruments, Martinsried, Germany).

### Staining of medial tract projection neurons in drones

PNs of the mALT were retrogradely stained by using methods described in detail by Kirschner et al. (2006), Zube et al. (2008) and Rössler and Zube (2011). Head capsules were opened and glands and tracheae covering the brain were removed. The tissue between the vertical lobe of the MB and the AL was punctured with a fine glass electrode on the medial side, where mALT fibers run relatively close to the surface of the brain. A few crystals of either Microruby or Biotin Dextran were gently applied to the punctured tissue by means of a broken micropipette. Mass-staining of all AL output tracts was achieved by using a similar technique and application of dye crystals into the center of the AL. Mass-staining of AL glomeruli was performed by the cutting of the antennae and the application of a droplet of Microruby (about 3-5 % in distilled water) to the antennal nerve stump. In all cases, animals were kept for 4–6 h in the dark under moist conditions to allow the dye to be transported intracellularly.

### Single PN staining

For single PN staining, the glial sheath was gently removed from the AL with fine forceps. PN cell bodies were approached with a patch-clamp pipette under visual control by using an upright microscope (BX51WI, Olympus Imaging Europa) and a micromanipulator (Junior Unit, Luigs & Neumann). We employed the intracellular [(K-gluconate (110 mM), HEPES (25 mM), KCl (10 mM), MgCl2 (5 mM), Mg-ATP (3 mM), Na-GTP (0.5 mM), EGTA (0.5 mM), pH 7.2, 284 mOsm)] and extracellular [(NaCl (140 mM), KCl (5 mM), MgCl2 (1 mM), CaCl2 (2.5 mM), NaHCO3 (4 mM), NaH2PO4 (1.2 mM), HEPES (6 mM), glucose (14 mM), pH 7.4, 326 mOsm)] solutions of Palmer et al. (2013). For staining, 0.5-1 % Lucifer Yellow (L0259, Sigma-Aldrich Chemie, Steinheim, Germany) was added to the intracellular solution. Whole cell patch clamp recordings were established by using an Axon system (Axopatch 200B, Digidata 1440A, ClampEx, Molecular Devices, Oregon, USA) and the membrane voltage was kept at -60 mV.

### Neuranatomical analyses

Brains were placed in fixative solution (4 % formaldehyde) overnight, following the staining procedure and then rinsed five times for 10 min in PBS (phosphate-buffered saline, pH 7.2). Biotin-Dextran-injected brains were stained with Alexa-488-conjugated Streptavidin (S-11226, Molecular Probes, Eugene, Ore., USA) in PBS with 0.2% Triton X (1:125) for 48 h. Subsequently, brains were again rinsed five times for 10 min in PBS. Microruby- and Biotin-Dextran-labeled brains were dehydrated in an ascending ethanol series (50, 70, 90, 95, 2× 100 %, each 10 min) and cleared and mounted in methyl salicylate (M2047, Sigma-Aldrich Chemie) for confocal microscopy. Afterwards, brains were scanned with a confocal laser-scanning microscope (Leica TCS SP2; Leica Microsystems, Wetzlar, Germany). Images were processed with AMIRA 3.1.1 and 5.4 software (Mercury Computer Systems, Berlin, Germany) and AL glomeruli in one preparation were reconstructed by using the AMIRA wrapping tool. Image stacks were further processed with ImageJ 1.46j (Wayne Rasband, National Institutes of Health, Befesta, Md., USA). To count the glomeruli in all other successful mALT stainings (that were not three-dimensionally reconstructed in detail), we marked glomeruli in image stacks by using the segmentation editor implemented in ImageJ. Contrast and brightness were adjusted with GIMP 2.8.2 (GNU Image Manipulation Program, http://www.gimp.org) and images in figures were arranged with CorelDrawX6 (Corel, Ottawa, ON, Canada).

### Identification of glomeruli

After the successful staining of axonal projections, AL reconstructions in workers were mapped onto a template AL by using the VOI (volume of interest) method described in Kelber et al. (2006). The antennal nerve, the T1 tract and a specific glomerulus (A17; after Galizia et al. 1999) were used as landmarks. Additionally, one AL reconstruction of a mass-staining of ORN axons of the whole antennal nerve was mapped onto the same template AL. This reconstruction allowed the identification of the AL input tracts in the template AL. With the identified glomeruli and the input tracts in the template AL, the matching of sensilla-stained ALs allowed the assignment of axonal projections of ORNs to a specific input tract and to particular glomeruli.

### Statistics

Relative innervation frequencies of the input clusters (T1-T4) were analyzed with a Friedman analysis of variance (ANOVA) and a post-hoc Wilcoxon rank sum test with Bonferroni correction for multiple comparisons. The distribution of ORN axons in the various glomerular clusters originating from the staining of *S. basiconica*-rich regions was compared with a hypothetical distribution in the glomerular input clusters based on the natural distribution by using Fisher’s exact test. All statistic tests were performed with R 2.10.1 (R Foundation for Statistical Computing, Vienna, Austria).

## Results

### ORN axon projections from many *S. trichoidea* and *S. basiconica* are broadly distributed into AL glomeruli

**Fig. 2:**
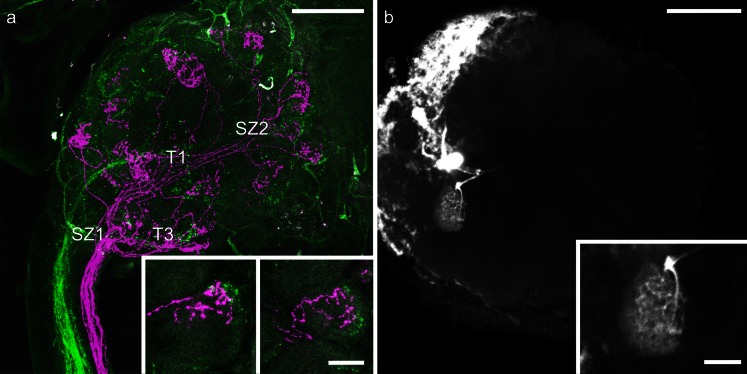
**a** Z-projection of a double-mass-staining of hair-like sensilla on segment 9 (*magenta*) and segment 4 (*green*) of honey bee antenna. The input tracts *T1* and *T3* are indicated. Note the two sorting zones (*SZ1*, *SZ2*). *Bar* 100 μm. *Insets* Detailed views of two glomeruli that are innervated by axons from olfactory receptor neurons from both the distal and the proximal parts of segment 9 in a layered fashion. *Bar* 25 μm. **b** Z-projection of an antennal lobe with an intracellularly stained projection neuron innervating a single glomerulus. *Bar* 100 μm. *Inset* Stained glomerulus in more detail; the dendritic arborizations of a projection neuron ramify throughout the entire glomerulus. *Bar* 25 μm

In the honeybee, ORN axons from *S. placodea* are broadly distributed across glomeruli of the AL and do not preferentially terminate in a specific sensory input cluster (Kelber et al. 2006). To determine whether this also held true for hair-like sensilla, we started with unselective mass-stainings of hair-like sensilla (*S. trichoidea* and *S. basiconica*) on a proximal (4th) and a distal (9th) segment of the same antenna by using two fluoresecent tracers. With this mass-staining method, no specific tract preferences of axons from ORNs housed in hair-like sensilla was found. However, the trajectories of axonal projections revealed a conspicuous axon-sorting-zone-like region at the AL entrance in which axons from the two stained axon bundles in the antennal nerve intermingled (Fig. 2a). Similarly, at the end of the antennal sensory input tracts, ORN axons showed a typical crossing pattern before they diverged to form terminal arborizations in specific subcompartments of individual glomeruli (Fig. 2a). Several glomeruli were innervated by ORN axons from the distal and the proximal segments. Interestingly, ORN axons from different antennal segments arborized in different layers within the same glomerulus (Fig. 2a, b). The dendritic arborizations of a single PN, in contrast to ORN axons, ramified across the entire volume of a glomerulus (Fig. 2b).

### Axons from ORNs in *S. trichoidea* of a single antennal segment arborize in the T1, T2 and T3 cluster of glomeruli

**Fig. 3:**
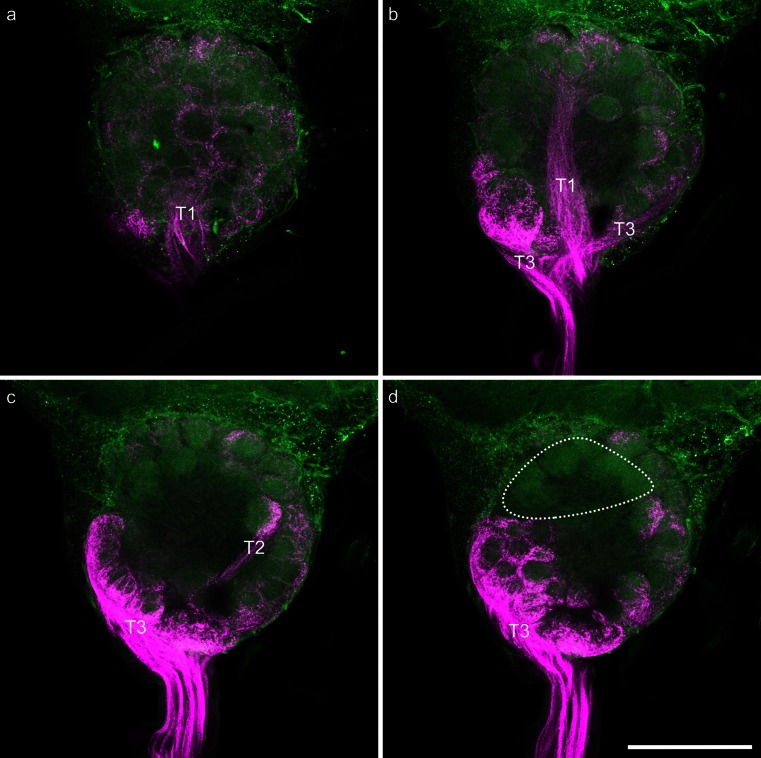
Images of a representative mass-staining of the axons of olfactory receptor neurons in *Sensilla trichoidea* of segment 5 of the honeybee worker antenna. Axons of olfactory receptor neurons are shown in *magenta*; the background was visualized via autofluorescence of the tissue and is shown in *green*. **a** Z-projection of 20 μm of the dorsal part of the stained antennal lobe; the glomeruli innervated from T1 input tract (*T1*) are clearly visible. **b** Z-projection of 20 μm of the dorsal middle part of the stained antennal lobe. Glomeruli innervated from the T3 input tract (*T3*) and the T1 input tract (*T1*) are clearly visible. **c** Z-projection of 20 μm of the ventral middle part of the stained antennal lobe. Glomeruli innervated from the T3 input tract (*T3*) and the T2 input tract (*T2*) are clearly visible. **d** Z-projection of 20 μm of the ventral part of the stained antennal lobe. Glomeruli innervated from the T3 input tract (*T3*) are clearly visible; the only non-innervated glomeruli (probably associated with T4 input tract) are indicated by the *dashed circle*. *Bar* 200 μm

As we found no specific preference of ORN projections for a certain glomerular cluster with unselective mass-staining of hair-like sensilla on different antennal segments, we set out to focus on the staining of ORNs from *S. trichoidea* only. Selective mass-labeling of axons from ORNs in *S. trichoidea* in honeybee workers was performed by cutting sensilla only at the middle region of the fifth antennal segment (Fig. 1). This mass-labeling technique excludes *S. basiconia*, which are located at the segment borders only but might well contain some mechanosensitive *S. trichoidea* (Lacher 1964) in addition to the olfactory sensilla. This staining (*n* = 13) revealed bundles of ORN axons terminating in the AL; a typical example is shown in Fig. 3. The trajectories of labeled ORN axons clearly changed from a more or less parallel organization into sorting-zone-like crossing patterns close to the entrance to the AL. From there, axonal projections proceeded into three tracts (T1, T2, T3) leading to the associated glomerular clusters of the AL. The T4 input tract and the associated glomerular cluster were not stained in any of the 13 preparations (Fig. 3d). A few axons proceeded into the dorsal lobe indicating that some of the stained sensilla stained were mechanosensory in nature (data not shown).

### Axons from ORNs in *S. basiconica* preferentially innervate the T3 cluster of glomeruli

**Fig. 4:**
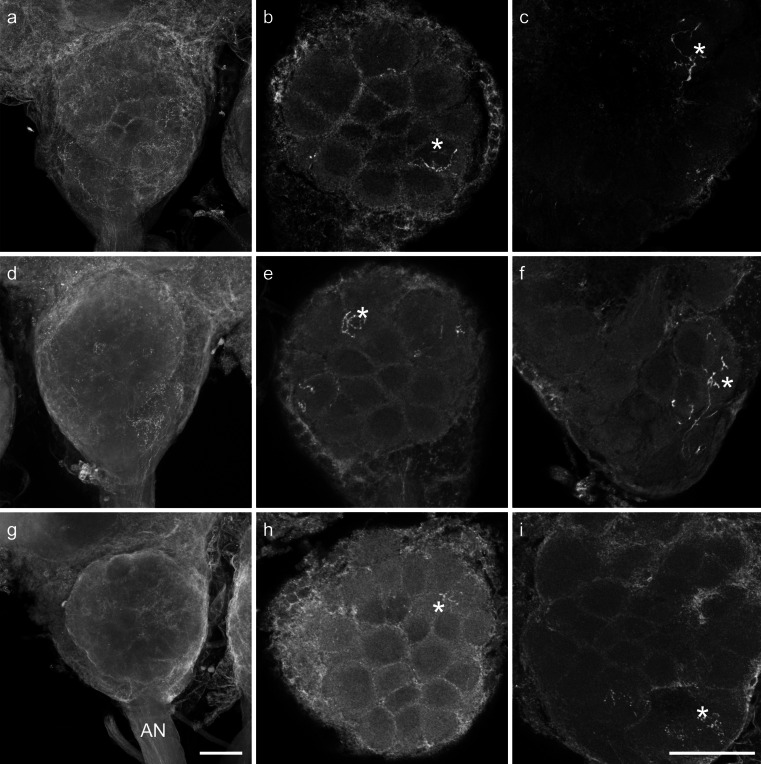
Confocal image stacks from two antennal lobes after staining of *Sensilla basiconica*-rich regions of the antenna (**a–f**) and image stacks of a single-sensillum staining of a *S. basiconicum* (**g–i**). Stained glomeruli (*asterisks*). **a**, **d**, **g** Complete stacks of antennal lobes with the antennal nerves (*AN*) and olfactory receptor neuron (ORN) arborizations in single glomeruli can hardly be identified in the complete image stacks. *Bar* 100 μm. **b**, **e**, **h** Substacks of the antennal lobes with identifiable ORN innervation in individual glomeruli in the T1 glomerular cluster. **c**, **f**, **i** Substacks of the antennal lobe with identifiable ORN innervation in individual glomeruli in the T3 glomerular cluster region. *Bar* 100 μm

**Fig. 5:**
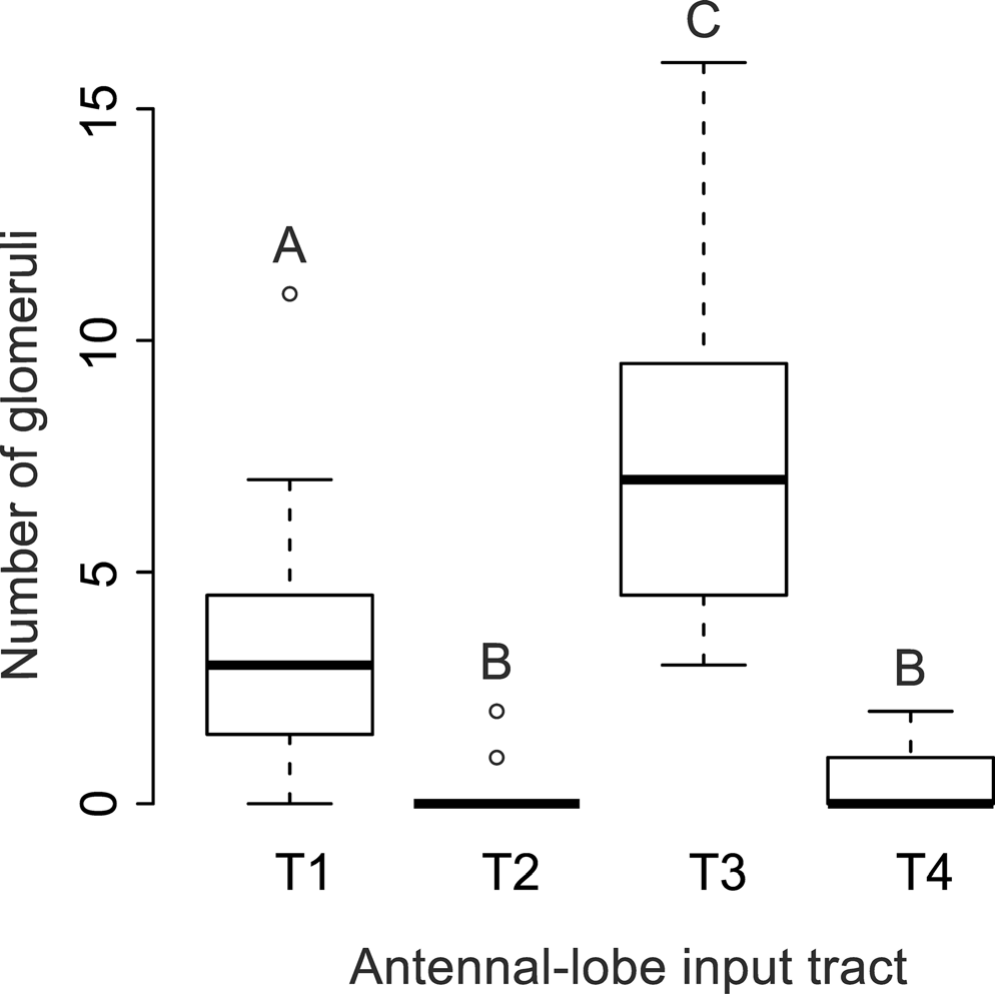
Numbers of glomeruli containing axonal projections from selective staining of ORNs in multiple *Sensilla basiconica*. Antennal lobe input tract T3 glomeruli were stained significantly more often compared with T1 glomeruli and T1 glomeruli were stained more often than T2 and T4 glomeruli (Friedman ANOVA, post-hoc Wilcoxon rank sum test with Bonferroni correction for multiple comparisons, *n* = 15, *A–C* significant differences between clusters)

As *S. basiconica* are far less numerous compared with *S. trichoidea*, a preference of axons from ORNs housed in *S. basiconica* for a certain input cluster might be masked in the mass-staining of hair-like sensilla. We therefore selectively stained sensilla in *S. basiconica*-rich regions; this was successful in 15 worker bees. In all cases, axonal branches of stained ORN axons were mainly restricted to the outer regions of individual glomeruli (Fig. 4). Glomerular borders were visualized by autofluorescence. Between 3 and 29 glomeruli per bee were stained in the different preparations. All stained glomeruli were mapped into the template AL and assigned to their input-tract glomerular clusters. In 13 out of 15 bees, T3-associated glomeruli were the most frequently stained glomeruli. In total, 180 ORN axons were stained in 15 bees; 57 projected to T1, four to T2, 111 to T3 and eight to T4 (Fig. 5; exact numbers of stained glomeruli can be found in supplementary Table 1). In one case, we were able to determine, by visual inspection, that the staining was from only a single *S. basiconicum* that had been cut (Fig. 4g-i). In this case, a single glomerulus was labeled in the T1 cluster and nine glomeruli were innervated in the T3 cluster. As *S. trichoidea* were also present in the segmental border regions of the antennae enriched with *S. basiconica*, we could not completely exclude that some *S. trichoidea* were also stained in most of the other group stainings of sensilla in the *S. basiconica*-enriched regions. To test this statistically, we calculated the relative innervation frequencies for each specimen. We compared these frequencies with a Friedman ANOVA (*P* = 5.28*10^-8^, chi^2^ = 36.72, *n* = 15) and observed differences between at least two groups within the data. A post-hoc Wilcoxon rank sum test with Bonferroni correction for multiple comparisons revealed T3 as the most frequently stained cluster of glomeruli. Significant differences were found between all clusters, except between T2 and T4 (Fig. 5, exact *P*-values: T1:T2 4.9 × 10^-4^ T1:T3 6.3 × 10^-4^ T1:T4 2.3 × 10^-3^ T2:T3 9.5 × 10^-6^ T2:T4 1.0 T3:T4 1.5 × 10^-5^). As the distribution of glomeruli among the input clusters is not homogenous (Flanagan and Mercer 1989; Galizia et al. 1999), we calculated a hypothetical distribution of glomeruli (T1: 79, T2: 8, T3: 85, T4: 8) by using the total number of glomeruli (180) stained in our experiments. Use of Fisher’s exact test between the hypothetical distribution of glomeruli and the observed distribution revealed a significant difference between the two distributions (*P* = 0.032). When we tested only the two major glomerular clusters (T1 and T3), the difference between the expected and the observed distribution had an even higher significance level (*P* = 0.0076). The projection patterns of sensilla in *S. basiconica*-enriched zones of the antenna, therefore, further indicate that axons of ORNs housed in *S. basiconica* preferentially, although not exclusively, project to the T3 cluster of olfactory glomeruli in the AL of worker bees.

### Number of glomeruli innervated by mALT projection neurons is reduced in honeybee drones

**Fig. 6:**
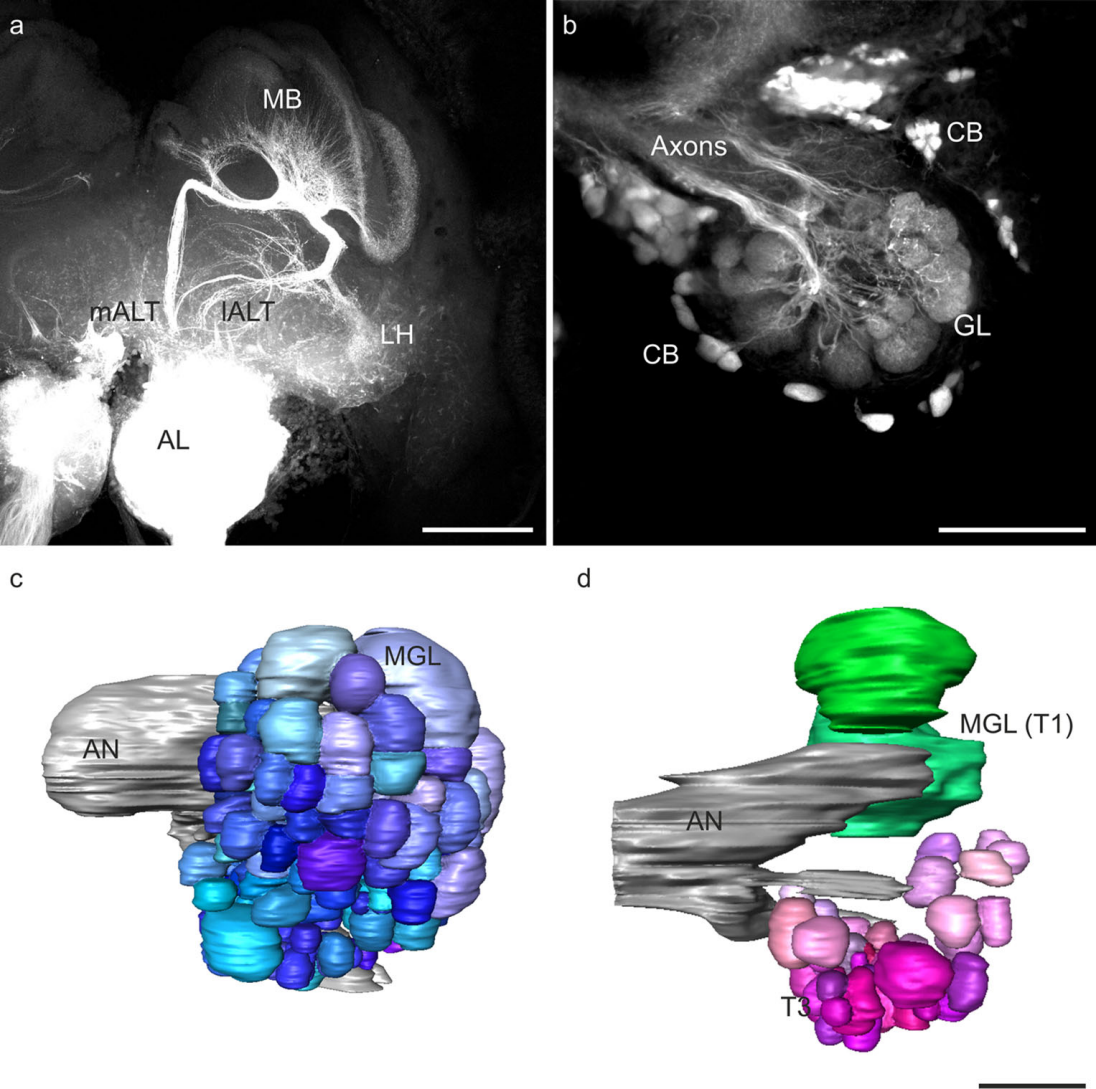
**a** Z-projection of a mass-staining of antennal lobe (*AL*) projection neurons in the honeybee drone; the AL, the medial and the lateral AL tract (*mALT*, *lALT*) and arborizations in the mushroom bodies (*MB*) and the lateral horn (*LH*) are visible. *Bar* 200 μm. **b** Z-projection of mALT staining in a honeybee drone. The axons, cell bodies (*CB*) and dendritic glomerular (*GL*) innervation are visible. *Bar* 100 μm. **c** Three-dimensional reconstruction of a drone AL with the antennal nerve (*AN*) after staining of all olfactory receptor neuron axons. Two macroglomeruli (*MGL*) are indicated. **d** Reconstruction of the mALT proportion of glomeruli and two macroglomeruli (*MGL*) within the T1 glomerular cluster (*T1*) as landmarks in a drone AL. The two lALT-associated MGL are shown in *green*, whereas mALT glomeruli in the T3 glomerular cluster (*T3*) are shown in shades of *magenta*. *Bar* 100 μm

**Fig. 7:**
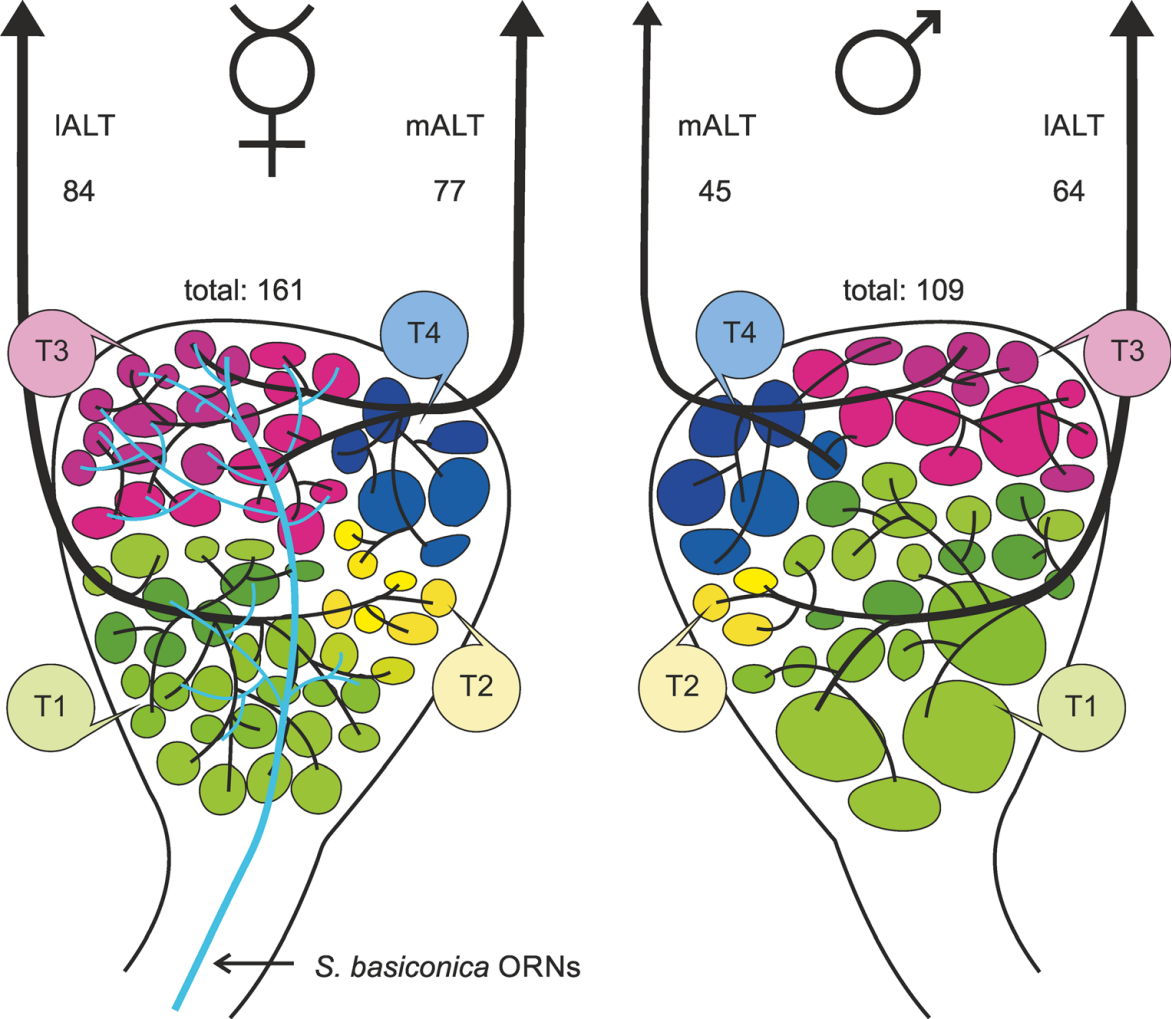
Representations of a honeybee worker (*left*) and drone (*right*) antennal lobe (AL). Olfactory receptor neuron axons housed in *S. basiconica* are only present in workers and project via the antennal nerve mainly to the T3 glomerular cluster (*T3*) in the AL (*light blue projections*). The T1 glomerular cluster (*T1*) is drawn in *shades of green*, the T2 in *shades of yellow* (*T2*) and the T4 cluster in *shades of blue* (*T4*). The medial AL tract (*mALT*) innervates mainly T3 glomeruli and the lateral AL tract (*lALT*) mainly T1 glomeruli. The T3 glomerular cluster and the mALT output tract are reduced in drones. The estimated numbers of glomeruli innervated by mALT and lALT projection neurons and the total numbers of glomeruli in the female (from Kirschner et al. 2006) and male AL are indicated (*ORNs* olfactory receptor neurons)

We have shown that ORNs from *S. basiconica* preferentially project to the T3 glomerular cluster in honeybee workers. This glomerular cluster is however reduced in honeybee drones (Nishino et al. 2009) and is associated with the mALT in honeybee workers (Kirschner et al. 2006). Thus, we assumed that the mALT should also be reduced in drones. Anterograde fluorescent staining of the output tracts of the AL did not reveal any obvious differences in tract diameters between the mALT and lALT in confocal images (Fig. 6a). In drones, as in workers, mALT PNs project via the MBs to the LH, whereas lALT PNs project first to the LH and then to the MBs (Fig. 6a). One preparation with a mass-staining of ORN axons in drones was used to reconstruct all glomeruli of the AL (Fig. 6c). This revealed a total of 109 glomeruli (107 glomeruli were counted in a second mass-staining). Specific staining of the mALT was used to determine the number of mALT glomeruli. One backfill of the mALT PNs (Fig. 6b) was reconstructed (Fig. 6d) and revealed a total of 45 glomeruli innervated by mALT PNs. In the remaining successful mALT labelings, a similar or even smaller number of glomeruli was counted (numbers from all successful mALT stainings: specimen 1: 39, specimen 2: 45, specimen 3: 45, specimen 4: 39, specimen 5: 43, *n* = 5). For further calculations, we used the highest number of labeled glomeruli, as some glomeruli might not have been stained by retrogradely labeling the PN arborizations in the AL. In honeybee workers, ~920 PNs innervate ~161 glomeruli (Rybak 2012), resulting in a hypothetical ratio of 5-6 PNs per glomerulus. No data on the number of PNs in drones are available but by assuming multiple PNs per glomerulus and by considering the fact that only one PN needs to be stained to identify a glomerulus, we assumed that the highest numbers of glomeruli counted in our results were representative of the total number of mALT innervated glomeruli. Subtraction of the average number of maximally counted mALT-associated glomeruli (45) from the total number of 109 glomeruli revealed an estimated number of 64 lALT-associated glomeruli (numbers in Fig. 7). Compared with the situation in females (~161 glomeruli in total; Kirschner et al. 2006), this indicates that most of the glomeruli missing in drones can be assigned to the mALT hemilobe of the AL. In total, 24 % of the lALT glomeruli and 42 % of the mALT glomeruli were absent in drones compared with workers. Whereas the mALT to lALT ratio of the AL in females is roughly 1:1, only ~39 % of all AL glomeruli in drones were associated with the mALT compared with ~61 % glomeruli associated with the lALT. The combination of our present results with the data from Kirschner et al. (2006) indicates that ORNs from *S. basiconica* in female worker bees preferentially innervate the T3 cluster of glomeruli and that this mALT-innervated cluster of glomeruli is reduced in drones (Fig. 7).

## Discussion

This study shows that axons of ORNs from *S. basiconica*, which are absent on drone antennae, mainly project into the T3 cluster of glomeruli in the AL of worker bees (Fig. 7). The T3 cluster of glomeruli has been demonstrated to be reduced in drones (Nishino et al. 2009) and our results reveal that mALT PNs (which, in the female AL, receive input from the T3 cluster) innervate fewer glomeruli in drones compared with workers. This indicates that information from the *S. basiconica* is associated with the mALT pathway in females and further suggests that this part of the mALT pathway is reduced in drones compared with workers. A similar reduction of the mALT-associated portion of AL glomeruli has been shown in males of the ant *Camponotus floridanus* (Zube and Rössler 2008).

### General features and sorting of axonal projections of ORNs

The trajectories of individual axons in the mass-stainings indicate that ORN axons are guided to their glomerular targets via a two-step sorting process, one at the segregation into T1-T4 at the entrance of the antennal nerve and a second one at the end of the tracts, just before the axons diverge to their glomerular targets. We show that ORN axons from the various antennal segments run in parallel along the antennal nerve until they reach the AL entrance. This is analogous to the ORN axon-sorting zone in the moth *Manduca sexta* (Rössler et al. 1999). After the initial rearrangements at the AL entrance, however, axonal projections in the honeybee proceed again in parallel along the tracts (T1-T4) until they reach the glomerular clusters. At these glomerular clusters, ORN axons appear to be rearranged in another crossing pattern until they terminate in distinct layers within individual glomeruli. This indicates that the organization in the four olfactory sensory tracts in the honeybee involves a more complex sorting process compared with the single sorting zone at the AL entrance shown in moths (Rössler et al. 1999). This process requires further clarification in future developmental studies.

Within individual glomeruli, ORN axons of sensilla from the distal and proximal segments of the antenna terminate in different layers (Fig. 2). These projection patterns indicate a change from the topographic position of sensilla on the antenna to the spatial organization of ORN target fields within single AL glomeruli. In contrast to the ORN projections, PN dendrites might arborize throughout the entire glomerulus (Fig. 2c, d; see also Müller et al. 2002; Krofczik et al. 2008) indicating that individual PNs receive convergent input from ORNs from widely separated topographical locations that innervate the same glomerulus.

### Glomerular targets of ORN axons from *S. trichoidea*

Selective mass-staining of *S. trichoidea* labels bundles of ORN axons projecting to the AL (Fig. 3). The trajectories of ORN axons can be followed along three tracts (T1, T2, T3; Fig. 3). In none of the 13 preparations have we found staining of T4 glomeruli (Fig. 3d). Interestingly, despite a wide distribution of axons in almost all glomeruli of the AL, slightly brighter staining has been detected in T3 glomeruli compared with T1 glomeruli. Whether this is attributable to a higher density and/or brighter labeling of individual axons cannot be resolved with our method. ORN axons from *S. placodea* have also been shown to project into all four glomerular clusters in the honeybee (Kelber et al. 2006). For a more detailed analysis, single fills of *S. trichoidea* ORNs would be necessary. Our staining demonstrates that ORN projections from *S. trichoidea* from only one antennal segment are widely distributed across three glomerular clusters (T1-T3) that contain >95 % of all AL glomeruli in the honeybee (Kirschner et al. 2006). Assuming that this is true for all antennal segments (we found similar results for segments 3, 5, 6, 7 and 8), this implies a high redundancy of the antennal segments within individual glomeruli. This ensures that the antenna efficiently captures sufficient odor molecules for odor identification but rather excludes a topographical resolution of odor reception (e.g., ORNs at the proximal or distal part of the antenna). In contrast to ORNs, PNs have dendritic arborizations across entire glomeruli (Fig. 2c, d). Whether the position of ORN projections in a distinct layer of the glomerulus (and its position on the antenna) in the honeybee has any physiological impact on PN responses, however, needs to be investigated in physiological recordings.

### Projection patterns of ORN axons from *S. basiconica*

Mass-staining techniques of hair-like sensilla on entire antennal segments have not allowed us to resolve glomerular cluster preferences of *S. basiconica*-associated ORN axons, as even the *S. basiconica*-rich segment 9 contains over 10× more *S. trichoidea* than *S. basiconica* (Esslen and Kaissling 1976). To increase the proportion of *S. basiconica*-associated ORNs, we selectively stained hair-like sensilla in the distal regions of individual antennal segments that show a ring-like arrangement of *S. basiconica* (Fig. 1). These experiments revealed that ORN axons from these sensilla terminate to a significantly higher proportion in glomeruli of the T3 cluster of AL glomeruli (Figs. 5, 7). In these staining experiments, we have preferentially aimed to cut *S. basiconica* but because of the small size of these sensilla in the honeybee, staining of *S. trichoidea*, in most cases, cannot be completely excluded. Therefore, we used a statistical method to analyze cluster preference in these selective stainings. Furthermore, the approved staining (by visual inspection) of a single *S. basiconicum* has shown that nine of the T3 glomeruli and only one T1 glomerulus are innervated thereby supporting the multiple sensilla staining results. The single *S. basiconicum* stained in our experiments comprised 10 ORNs. This is in agreement with results from histological investigations showing that *S. basiconica* in the honeybee contain 8-12 ORN dendrites (Nishino et al. 2009). In other Hymenoptera, ORN numbers in *S. basiconica* have been demonstrated to range between 30-40 ORNs in wasps (Lacher 1964) and 3-53 ORNs in ants (Kelber et al. 2010). Until now, the notion that *S. basiconica* are indeed olfactory sensilla is mainly based on morphological criteria. Whereas the morphological evidence is strong, however, clear physiological evidence is still lacking. Recordings from *S. basiconica* have only been mentioned as unpublished results in Akers and Getz (1993). Our anterograde staining of the axonal projections of ORNs housed in *S. basiconica* has revealed innervations of relatively high numbers of preferentially T3 glomeruli in the AL. This adds further evidence to the assertion that *S. basiconica* are olfactory sensilla (Slifer and Sekhon 1961; Lacher 1964). Our results suggest that axons of *S. basiconica*-associated ORNs preferentially project to glomeruli of the T3 cluster and only partially to glomeruli of the T1 cluster.

### Differences between honeybee drones and workers and potential functional consequences

Interestingly, *S. basiconica* are present in honeybee workers and queens but not in drones (Esslen and Kaissling 1976). The absence of *S. basiconica* in males seems to be a typical feature in Hymenoptera including the honeybee, bumblebees, solitary bees and ants (Ågren 1977; 1978; Wcislo 1995; Ågren and Hallberg 1996; Nakanishi et al. 2009, 2010; Mysore et al. 2010; Nishikawa et al. 2012; Streinzer et al. 2013). Wcislo (1995) argued that drones have reduced the number of a certain type of olfactory sensilla to increase their capabilities for pheromone detection and thereby to increase their probability for encountering a queen faster than other drones. The finding that drones have extremely high numbers of pore plates (*S. placodea*), which also house the pheromone-specific sensilla (Kaissling and Renner 1968), supports this view. Three of the enlarged glomeruli (macroglomeruli) are located in the T1 cluster of glomeruli (Nishino et al. 2009) and calcium-imaging studies have shown that components of the queen mandibular pheromone are processed in one of these enlarged glomeruli (Sandoz 2006). The processing of pheromone information in macroglomeruli is well known from sex pheromone communication in various species of moths (e.g., Christensen and Hildebrand 1987; Anton and Hansson 1994; Greiner et al. 2004) and has also been shown for an enlarged glomerulus which processes components of the trail pheromone in large leaf-cutting ant workers (Kleineidam et al. 2005; Kelber et al 2009; Kuebler et al 2010). The reduced number of *S. trichoidea* and the complete absence of *S. basiconica* and the associated neuronal pathway in the AL of drones might allow higher capacities for queen-pheromone-specific sensilla and a larger sex-pheromone-processing neuronal circuitry in the AL (Fig. 7).

### Female-specific olfactory subsystems and their possible function

We have been able to demonstrate that the reduction of glomeruli in the AL of honeybee drones is higher in the mALT- compared to the lALT-innervated hemilobe of the AL. ORN axons in the glomeruli of the T3 cluster are innervated by mALT PNs in females (Kirschner et al. 2006). This cluster is reduced in drones, whereas the T1 cluster is less reduced compared with that in the female AL. As drones lack *S. basiconica*, the reduction of the mALT-associated parts of the T3 glomeruli is likely to be related to the absence of ORNs from the *S. basiconica*. As three enlarged glomeruli (macroglomeruli, as indicated in Fig. 7) are present in the T1 cluster in drones (Sandoz 2006; Nishino et al. 2009), the slight reduction in the number of T1 glomeruli in drones might favor the macroglomeruli.

Several physiological studies, so far, have shown that no part of the honeybee AL and therefore neither the mALT or the lALT, is selectively specialized for either only social or floral odorants (Abel et al. 2001; Brill et al. 2013; Carcaud et al. 2012; Galizia et al. 2012; Krofczik et al. 2008; Müller et al. 2002; Rössler and Brill 2013; Yamagata et al. 2009). However, as honeybees are exposed to an enormous odor space in their natural environments, more odorants, in particular social (colony) cues and pheromones, remain to be tested in more detail and might give further indications concerning selective physiological properties and the molecular receptive range of *S. basiconica* ORNs. The ants *Camponotus floridanus* (Zube and Rössler 2008), *Camponotus japonicus* (Nishikawa et al. 2012) and *A. vollenweideri* (Kelber et al. 2010) have similarly been demonstrated to have a reduced number of glomeruli in males compared with females. Kelber et al. (2010) investigated projection patterns of *S. trichoidea* and *S. basiconica* ORNs in *A. vollenweideri*. Here, the ants possess six AL glomerular clusters (T1-T6) and *S. trichoidea* ORNs have been shown to project to all of them. Up to five different glomerular clusters have been found to be innervated by ORNs from a single *S. trichoideum*. In contrast, *S. basiconica* ORNs only projected to the T6 cluster (Kelber et al. 2010). In honeybee workers, we were able to reveal that *S. basiconica* preferentially project into the T3 cluster. *C. japonicus* ALs comprise seven glomerular clusters (T1-T7) and the glomerular cluster T6 is also worker-specific, similar to the T6 cluster in *A. vollenweideri* (Kelber et al. 2010; Nishikawa et al. 2012). Furthermore, here the T6 output neurons were demonstrated to project to specific subregions within the MBs and the LH and the authors speculate that the input to these glomeruli origins from *S. basiconica*-associated ORNs (Nishikawa et al. 2012). Nishikawa et al. (2012) argued that these brain regions are likely to be involved in social tasks in *C. japonicus*, as drones do not need to fulfil extensive social duties within the colony.

Another important aspect is that in several solitary bee species, the males also lack *S. basiconica* (Ågren 1977, 1978; Ågren and Hallberg 1996; Galvani 2012; Streinzer et al. 2013). In parasitoid wasps, cuticular hydrocarbon profiles have been shown to vary between closely related species, between sexes and according to the developmental environment (Khidr et al. 2013). Better detection abilities for cuticular hydrocarbons could serve females in kin detection and thus help them to avoid insemination by related males or even by males from other species. Another major distinction between drones and workers in both ants and social bees is that drones do not forage. This implies the involvement of *S. basiconica* in the detection of floral odorants. These studies show that various hymenopteran species possess female-specific olfactory subsystems consisting in specific sensilla, their respective ORNs and downstream odor-processing brain structures. The separation of these subsystems from the sexual isomorphic structures can be more (*A. vollenweideri*, *C. japonicus*) or less (*A. mellifera*) pronounced. Thus, variations in olfactory tasks and structures between males and females might differ across species.

Based on these differences described so far with regard to behaviors between male and female honeybees, we conclude that the *S. basiconica* might equally well be specialized for flower odorant detection or the detection of social olfactory cues or might even be generalistic.

### Concluding remarks

Honeybee workers possess a specific olfactory subsystem comprising *S. basiconica*, parts of the T3 cluster of glomeruli and a significant proportion of mALT PNs. Drones completely lack this olfactory subsystem (Fig. 7) and, additionally, have far fewer *S. trichoidea*, all features that might favor a more elaborated queen-pheromone-processing system and the associated higher numbers of *S. placodea* present in drones (Brockmann et al. 2006; Kaissling and Renner 1968; Sandoz 2006). At the behavioral level, drones, therefore, are likely to have more limited odor discrimination and recognition abilities compared with females. This limitation is likely to be associated with social (colony) odors and/or floral odors. The adaptation in drones for improved queen-pheromone detection including high numbers of *S. placodea* and pheromone-processing macroglomeruli is likely to increase mating probabilities. Honeybee workers, in contrast, are exposed to high selective pressure to identify and locate correctly a wide variety of odorants, including floral odorant mixtures, a large variety of pheromones and colony (social) cues. The different types of sensilla and the associated olfactory subsystems of glomeruli and output tracts in the AL appear to be well adapted for these tasks.

## Electronic supplementary material

Below is the link to the electronic supplementary material.

Supplemental Table 1 (DOCX 15 kb)
