# Supplementary material for: Olfactory subsystems in the honeybee: sensory supply and sex specificity
Source: Cell Tissue Res. 2014 May 13;357(3):583–95. doi: 10.1007/s00441-014-1892-y (PMC4148592; doi:10.1007/s00441-014-1892-y)
Supplement: Supplementary file 1 — (DOCX 15 kb) [file 441_2014_1892_MOESM1_ESM.docx]

Supplemental Table 1: Olfactory receptor neuron axon association to glomerular clusters after staining of *S.basiconica* rich regions. T1- T4 refers to the input tract glomerular clusters. The number of stained glomeruli and the relative frequency per specimen (calculated by dividing the number of glomeruli per tract by the total number of glomeruli stained in each specimen) is given.

| Specimen | T1 | T2 | T3 | T4 | ∑ |
| --- | --- | --- | --- | --- | --- |
| 1 | 2 (16.7%) | 0 (0%) | 10 (83.3%) | 0 (0%) | 12 |
| 2 | 11 (61.1%) | 0 (0%) | 6 (33.3%) | 1 (5.6%) | 18 |
| 3 | 0 (0%) | 0 (0%) | 3 (100%) | 0 (0%) | 3 |
| 4 | 11 (37.9%) | 0 (0%) | 16 (55.2%) | 2 (6.9%) | 29 |
| 5 | 7 (63.6%) | 0 (0%) | 4 (36.4%) | 0 (0%) | 11 |
| 6 | 3 (33.3%) | 2 (22.2%) | 4 (44.4%) | 0 (0%) | 9 |
| 7 | 4 (36.4%) | 0 (0%) | 6 (54.5%) | 1 (9.1%) | 11 |
| 8 | 1 (9.1%) | 0 (0%) | 10 (90.9%) | 0 (0%) | 11 |
| 9 | 4 (26.7%) | 0 (0%) | 11 (73.3%) | 0 (0%) | 15 |
| 10 | 0 (0%) | 0 (0%) | 4 (80%) | 1 (20%) | 5 |
| 11 | 3 (27.3%) | 0 (0%) | 8 (72.3%) | 0 (0%) | 11 |
| 12 | 5 (38.5%) | 1 (7.7%) | 5 (38.5%) | 2 (15.4%) | 13 |
| 13 | 3 (25 %) | 1 (8.3%) | 8 (66.7%) | 0 (0%) | 12 |
| 14 | 2 (20%) | 0 (0%) | 7 (70%) | 1 (10%) | 10 |
| 15 | 1 (10%) | 0 (0%) | 9 (90%) | 0 (0%) | 10 |
| total | 57 (31.7%) | 4 (2.2%) | 111 (61.7%) | 8 (4.4%) | 180 |
